# Livédo atypique et révélateur!?

**DOI:** 10.11604/pamj.2018.29.91.14517

**Published:** 2018-01-30

**Authors:** Mohamed El Amraoui, Naoufal Hjira

**Affiliations:** 1 Department of Dermatology-Venereology, Military Training Hospital Mohammed V, Rabat, Morocco

**Keywords:** Livédo, athérosclérose, membres inférieurs, Livedo reticularis, atherosclerosis, lower limbs

## Abstract

Atherosclerosis is a frequent and life-threatening complication in elderly patients with multimorbidity as well as with one or multiple cardiovascular risk factors. It can affect all the arterial trunks of the lower limbs and can cause obliterative arterial disease of the lower limbs. We here report the case of an elderly patient with ecchymotic livedo reticularis of the right thigh revealing ipsilateral obliterative arterial disease of the lower limb with septic loosening of total hip prosthesis. The study involved a 70-year old man with favorable atheromatous condition complicated 6 months before by ischemic heart disease revascularized with double prostheses (stent) and with right total hip prosthesis implanted 10 years before. He presented with large mesh and incomplete livedo reticularis associated with ecchymoses on the right thigh (A). Clinical examination showed reduced distal pulses in the right lower limb. Laboratory tests showed discrete inflammatory syndrome and normal haemostatic and immunological tests . Standard radiographic evaluation of the right hip showed a loosening of the distal end of the prosthesis (C). Echo-doppler of the lower limbs and angio-scanner showed occlusion of the right external iliac axis, right double primitive iliac stenosis and a lesion due to septic dislocation of the right femoral prosthesis with abscess collection measuring 48 mm/30 mm ( B). The patient was referred to the Division of Vascular Surgery for revascularization. Vascular imaging by echo-doppler and/or angio-scanner should be performed, in particular in elderly patients with livedo reticularis or venous collateral circulation and with cardiovascular risk factors.

## Image en médecine

L'athérosclérose est une fréquente et redoutable complication du sujet âgé multitaré et ayant un ou plusieurs facteurs de risque cardiovasculaires. Elle peut toucher tous les troncs artériels, au niveau des membres inférieurs elle est pourvoyeuse de l'artériopathie oblitérante des membres inférieurs. Nous présentons un cas de livédo ecchymotique de la cuisse droite chez un sujet âgé ayant révélé un artériopathie oblitérante du membre inférieur homolatéral avec un descellement septique d'une prothèse totale de la hanche. Un homme âgé de 70 ans, avec un terrain athéromateux favorable, compliqué il ya 6 mois d'une cardiopathie ischémique revascularisée par double prothèses (Stent) et ayant une prothèse totale de la hanche droite depuis 10 ans. A consulté pour un livédo à mailles larges et incomplètes associées à des ecchymoses de la cuisse droite (A). L'examen clinique a montré une diminution des pouls distaux du membre inférieur droit. Le bilan biologique montrait un discret syndrome inflammatoire et des bilans d’hémostase et immunologiques normaux. La radiographie standard de la hanche droite montrait un descellement du bout distal de la prothèse (C). L'écho-doppler des membres inférieurs et l'angio-scanner montraient une occlusion de l'axe iliaque externe droit, une double sténose iliaque primitive droite et une lésion de dislocation septique de la prothèse fémorale droite avec une collection abcédée de 48 mm/30 mm (B). Le patient a été référé à un service de chirurgie vasculaire pour revascularisation. Devant tous livédo ou une circulation veineuse collatérale chez un sujet surtout âgé et ayant des facteurs de risque cardiovasculaires, une imagerie vasculaire par écho-doppler et/ou angio-scanner s'avère très nécessaire.

**Figure 1:**
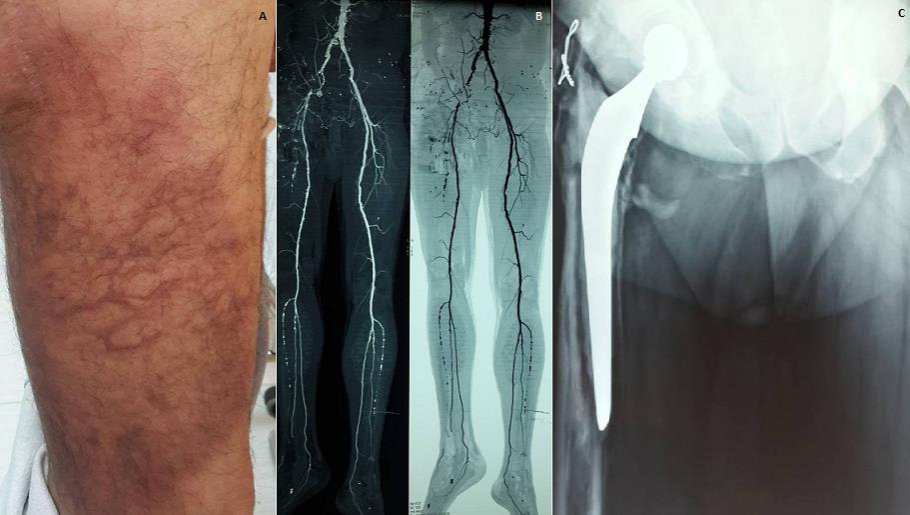
A) placard inflammatoire, écchymotique, avec livédo de la cuisse droite; B) radiographie standard de la hanche droite montrant un descellement de la prothèse totale de la hanche; C) angio-scanner montrant une occlusion de l’axe iliaque externe droit et une double sténose iliaque primitive droite

